# Tip-fixing underwater endoscopic mucosal resection without submucosal injection for a laterally spreading colon polyp

**DOI:** 10.1016/j.vgie.2025.03.034

**Published:** 2025-03-24

**Authors:** Koichi Okamoto, Tomoyuki Kawaguchi, Kaizo Kagemoto, Yoshifumi Kida, Yasuhiro Mitsui, Masahiro Sogabe, Yasushi Sato, Tetsuji Takayama

**Affiliations:** Department of Gastroenterology and Oncology, Institute of Biomedical Sciences, Tokushima University Graduate School, Tokushima, Japan

## Abstract

**Background and Aims:**

Underwater endoscopic mucosal resection (EMR) has become a popular endoscopic resection method for intermediate-to-large colorectal polyps. However, the snare tip can sometimes slip when opening or closing the snare, resulting in increased risk of piecemeal resection. To address this issue, we report our technique of tip-fixing underwater EMR without submucosal injection for a laterally spreading colon polyp.

**Methods:**

Degassed water was infused using a mechanical water pump to completely fill the lumen. By projecting the tip of the snare by 2 mm, a mucosal incision was made on the oral side of the lesion using a cutting current. The snare was positioned appropriately around the lesion. After the lesion was captured, resection was performed using electrocautery.

**Results:**

Complete en bloc resection was achieved with no adverse events, and the mucosal defect was completely closed using clips. Pathological findings indicated a low-grade tubulovillous adenoma with negative margins.

**Conclusions:**

In previous tip-in EMR studies, a spot-shaped mucosal incision was created at the oral normal mucosa with prior submucosal injections using the snare tip to fix the snare. However, submucosal injection was not required in our technique of tip-fixing underwater EMR. Essentially, intraluminal water serves as a heat sink which, when combined with a relatively thicker wall resulting from the relaxation of mucosal tension by removing intraluminal air, may protect against thermal injury of the deeper colonic wall even while making a precut with the snare tip. However, excessive snare exposure, overapplication of cautery, or deep snare driving could cause perforation, especially in thin-walled areas such as the right-sided colon or small intestine.

Underwater endoscopic mucosal resection (EMR) has become a popular endoscopic resection method for intermediate-to-large colorectal polyps.^1^^,^^2^ However, the snare tip can sometimes slip when opening or closing the snare, resulting in increased risk of piecemeal resection. To address this issue, we report our technique of tip-fixing underwater EMR without submucosal injection for a laterally spreading colon polyp (Fig. 1).

**Figure 1:**
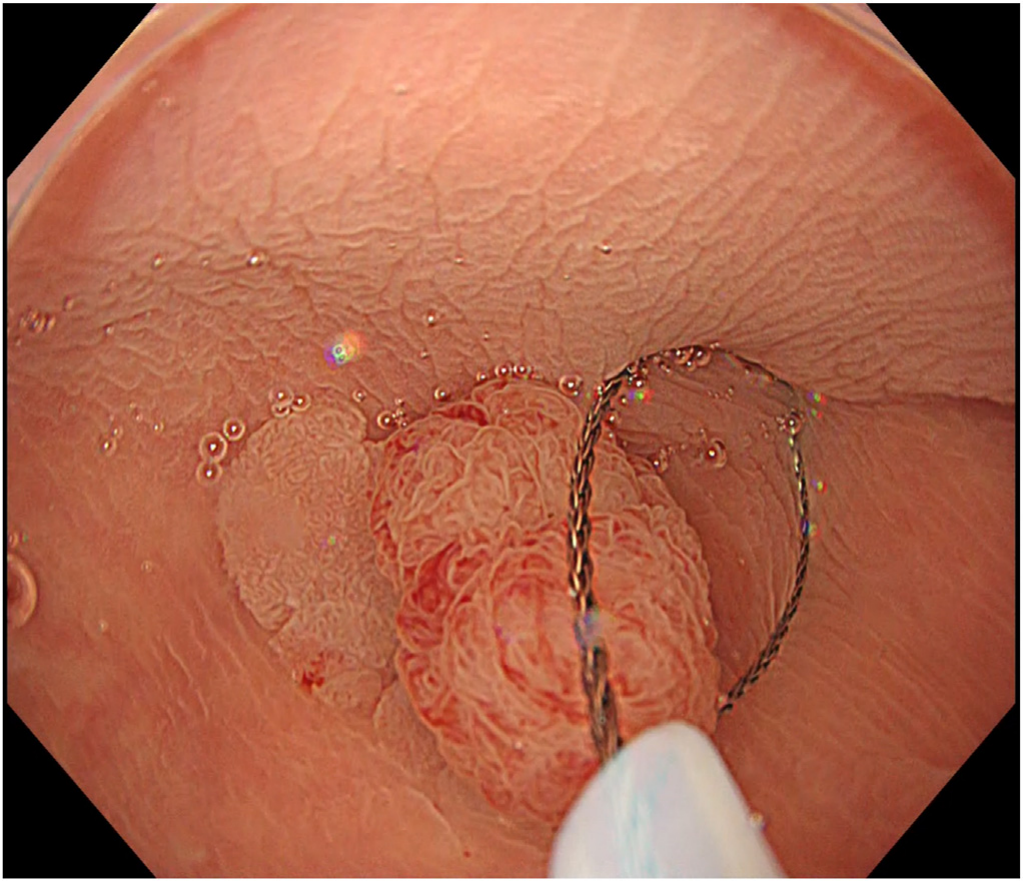
Representative endoscopic image of tip-fixing underwater endoscopic mucosal resection.

A 59-year-old woman with positive fecal occult blood test results underwent a colonoscopy, which revealed a laterally spreading tumor with a diameter of 20 mm in the sigmoid colon (Fig. 2). Magnifying endoscopy with narrow-band imaging showed regular microvessels and surface structures, suggesting an adenoma (Fig. 3). Therefore, we performed tip-fixing underwater EMR for this lesion (Video 1, available online at www.videogie.org).

**Figure 2:**
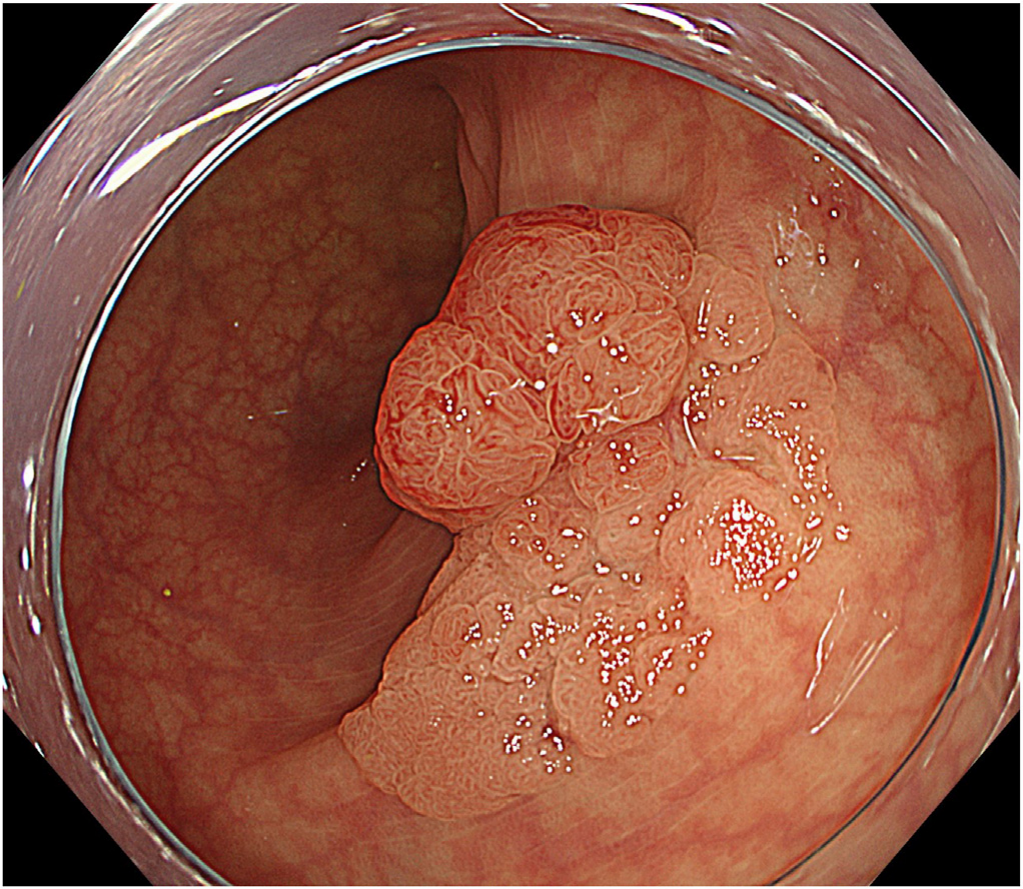
White-light image showed a laterally spreading tumor with a diameter of 20 mm in the sigmoid colon.

**Figure 3:**
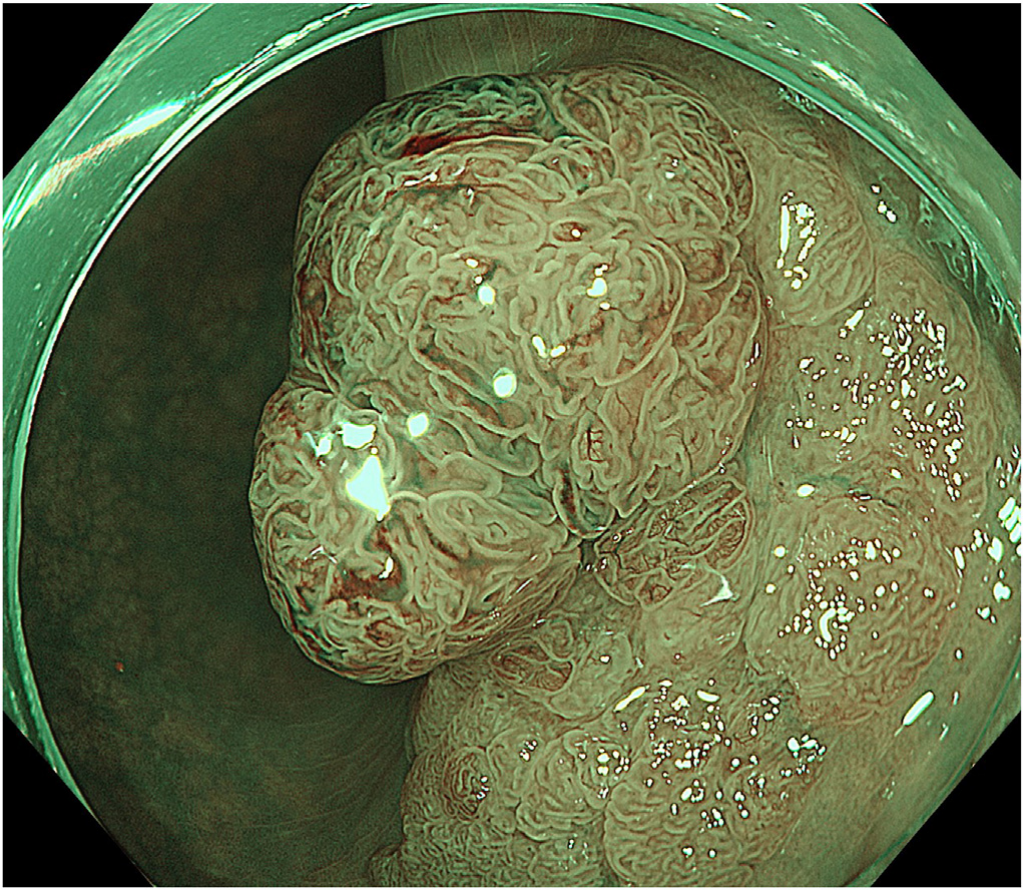
Magnifying endoscopy with narrow-band imaging showed regular microvessels and surface structures, suggesting an adenoma.

Degassed water was infused using a mechanical water pump (OFP-2; Olympus, Tokyo, Japan) (Fig. 4) to completely fill the lumen. By projecting the tip of the snare (SnareMaster; Olympus) by 2 mm, a mucosal incision was made on the oral side of the lesion using a cutting current (Endo-cut I, Effect 2; VIO3, Erbe, Tübingen, Germany) (Fig. 5). The snare was positioned appropriately around the lesion (Fig. 6). After the lesion was captured, resection was performed using electrocautery (Endo-cut I, Effect 2). Complete en bloc resection was achieved with no adverse events (Fig. 7), and the mucosal defect was completely closed using clips. Pathologic findings indicated a low-grade tubulovillous adenoma with negative margins.

**Figure 4:**
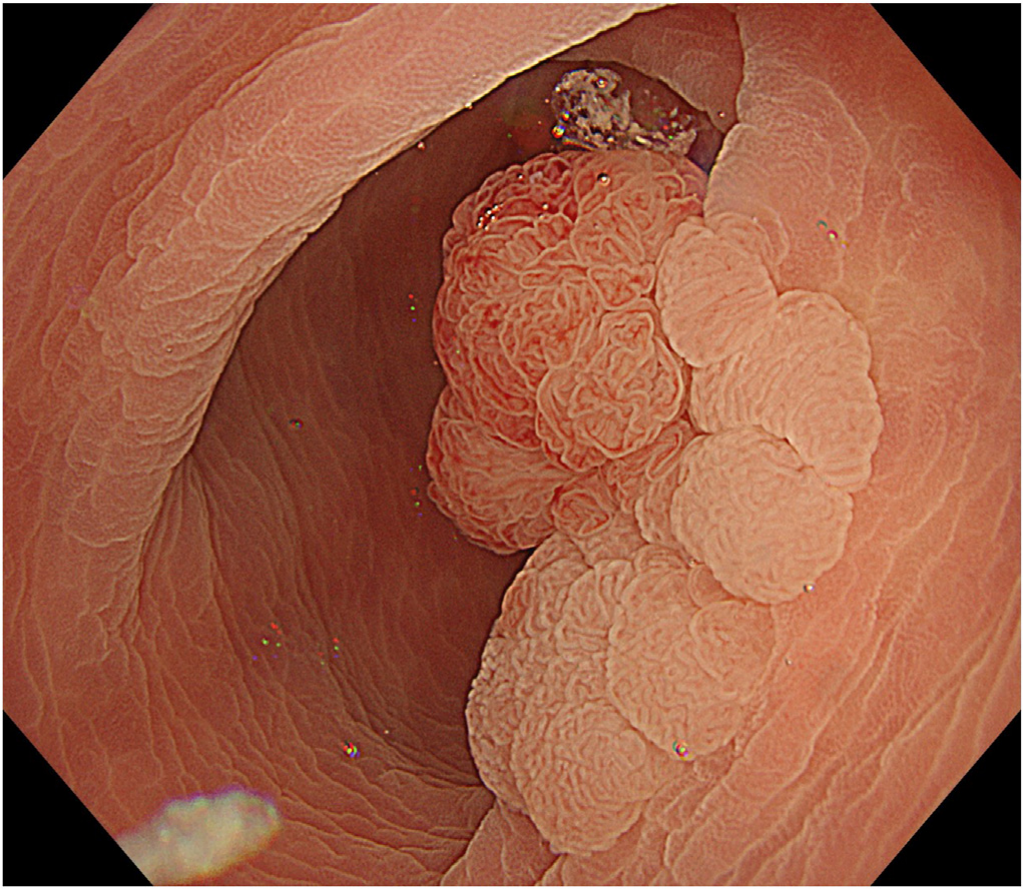
The lumen was filled with degassed water.

**Figure 5:**
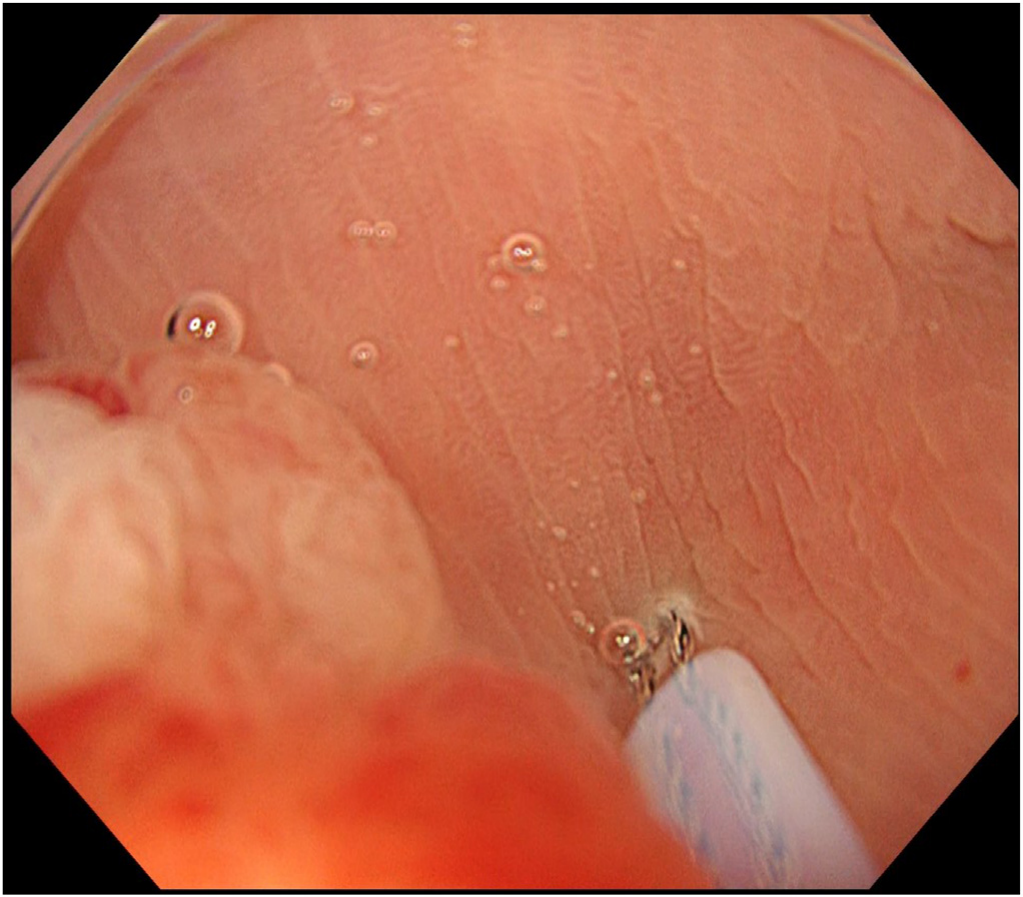
The distal edge of the lesion was cut using a cutting current without any submucosal injections.

**Figure 6:**
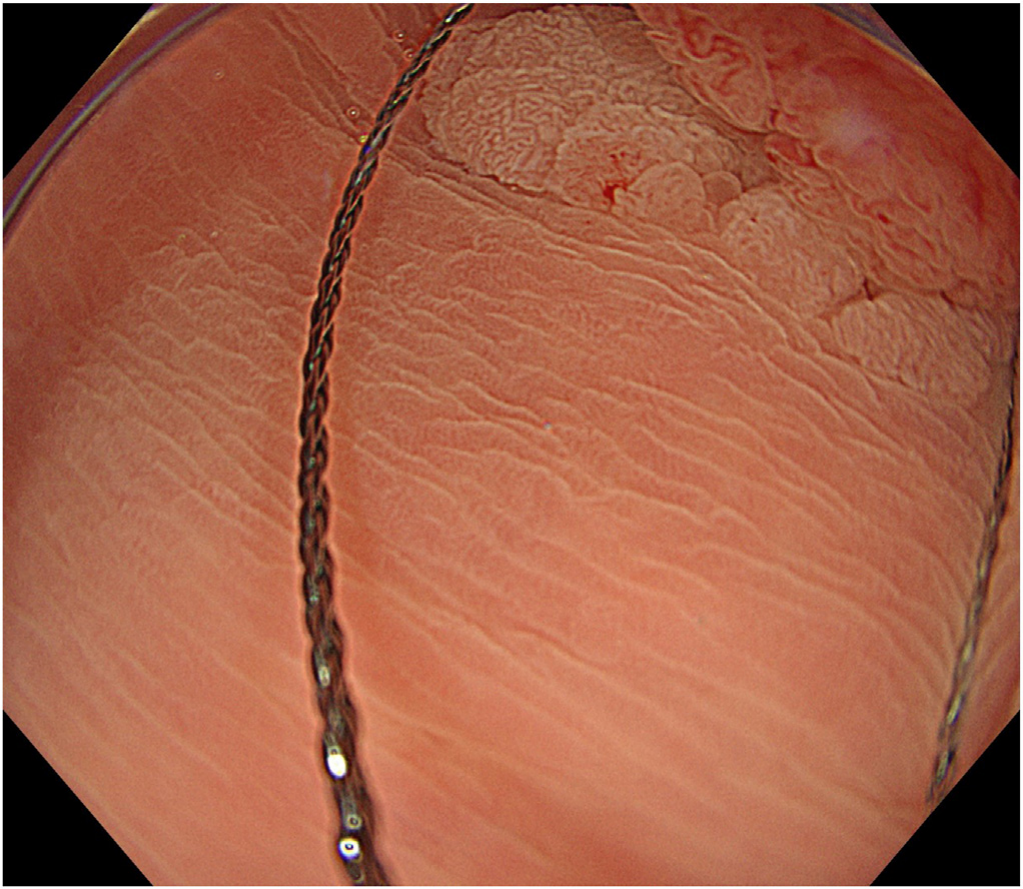
The snare was positioned around the lesion.

**Figure 7:**
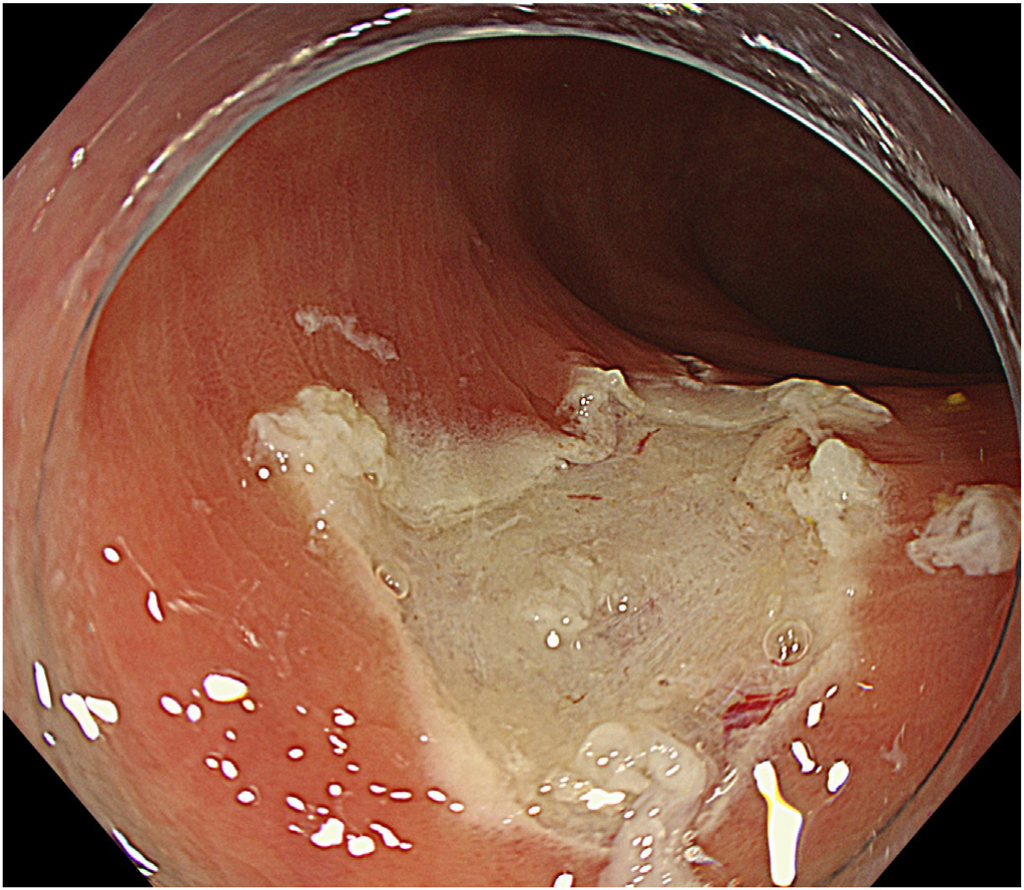
There was no residual lesion after resection.

In previous tip-in EMR studies, a spot-shaped mucosal incision was created at the oral normal mucosa with previous submucosal injections using the snare tip to fix the snare.^3^ However, submucosal injection was not required in our technique of tip-fixing underwater EMR. Essentially, intraluminal water serves as a heat sink which, when combined with a relatively thicker wall resulting from the relaxation of mucosal tension by removing intraluminal air, may protect against thermal injury of the deeper colonic wall even while making a pre-cut with the snare tip. In fact, we have reported that similar treatment can be performed for lesions in the duodenum.^4^ However, excessive snare exposure, overapplication of cautery, or deep snare driving could cause perforation, especially in thin-walled areas such as the right-sided colon or small intestine.

For colorectal polyps, this technique suits 10- to 20-mm lesions commonly indicated for underwater EMR, particularly when snare-tip slippage occurs or when poor distal visibility exists due to flexure location. Appropriate snare selection on the basis of lesion size is crucial.

## Patient consent

The patient in this article has given written informed consent to publication of the case details.

## Disclosure

All authors declare no conflict of interest.

## Supplementary data

Video 1 Degassed water was infused using a mechanical water pump. By projecting the tip of the snare by 2 mm, a mucosal incision was made on the oral side of the lesion using a cutting current. The snare was positioned appropriately around the lesion. After the lesion was captured, resection was performed using electrocautery. Complete en bloc resection was achieved.
